# Complete Genome Sequence of the Equol-Producing Bacterium *Adlercreutzia equolifaciens* DSM 19450^T^

**DOI:** 10.1128/genomeA.00742-13

**Published:** 2013-09-19

**Authors:** Hidehiro Toh, Kenshiro Oshima, Takehito Suzuki, Masahira Hattori, Hidetoshi Morita

**Affiliations:** Medical Institute of Bioregulation, Kyushu University, Higashi-ku, Fukuoka, Japana; Graduate School of Frontier Sciences, the University of Tokyo, Kashiwa, Chiba, Japanb; School of Veterinary Medicine, Azabu University, Chuo-ku, Sagamihara, Kanagawa, Japanc

## Abstract

*Adlercreutzia equolifaciens* DSM 19450^T^ was isolated from human feces and is able to metabolize daidzeins (soybean isoflavonoids) to equol. Here, we report the finished and annotated genome sequence of this organism.

## GENOME ANNOUNCEMENT

The potential health benefits of soybeans and soy foods have become increasingly recognized worldwide. Isoflavones are found abundantly in soy products. Because isoflavones are structurally similar to the female hormone estrogen, the associated health benefits are thought to be due to their abilities to bind to estrogen receptors. Among the isoflavonoids, equol, a metabolite of daidzein produced by members of the gut microflora, is thought to be the primary soy isoflavone derivative. *Adlercreutzia equolifaciens* strain DSM 19450^T^ was isolated from the feces of a healthy human and is able to metabolize daidzeins to equol (1).

The complete genome sequence of *A. equolifaciens* DSM 19450^T^ was determined by a whole-genome shotgun strategy with the Sanger method. Genomic libraries containing 3-kb inserts were constructed and sequenced, and 31,648 sequences were generated, giving 7.5-fold coverage from both ends of the genomic clones. Sequence reads were assembled with the Phred-Phrap-Consed program. The remaining gaps between the contigs were closed by direct sequencing of clones. Protein-coding gene prediction and annotation were performed as described previously (2). The genome of *A. equolifaciens* DSM 19450^T^ consists of a circular 2,862,526-bp chromosome containing 2,281 predicted protein-coding genes, and it has no plasmid. Of all the predicted protein-coding genes, 1,465 and 1,329 were also found in the genomes of *Eggerthella lenta* DSM 2243 and *Eggerthella* sp. strain YY7918, respectively (3, 4).

Shimada et al. (5) showed the model of the equol biosynthetic pathway starting from daidzein in *Lactococcus garvieae* 20-92, which is a lactic acid bacterium that produces equol from daidzein (5). The equol biosynthetic pathway consists of four enzymes [NADP(H)-dependent daidzein reductase, dihydrodaidzein reductase, tetrahydrodaidzein reductase, and dihydrodaidzein racemase], and the genome of *A. equolifaciens* DSM 19450^T^ also contains the four genes AEQU_2228, AEQU_2230, AEQU_2231, and AEQU_2234.

An interesting feature of the genome of *A. equolifaciens* DSM 19450^T^ is that it contains two unusually large genes (“giant” genes). The lengths of the open reading frames of AEQU_0093 and AEQU_1251 are 74,766 and 74,247 nucleotides, respectively. A previous report has shown that giant putative surface proteins exhibit a characteristic signature of amino acid usage, which is rich in aspartate, glutamate, threonine, serine, and asparagine and poor in lysine, arginine, and cysteine (6). The gene products of AEQU_0093 and AEQU_1251 showed a similar signature. Furthermore, both proteins have a C-terminal LPXTG motif that sortase enzymes recognize. Thus, these two giant genes may encode extracellular surface proteins.

### Nucleotide sequence accession number.

The sequence data for the *A. equolifaciens* DSM 19450^T^ genome are available from DDBJ/GenBank/EMBL under accession no. AP013105.
